# *TFE3*-rearranged renal cell carcinoma with osseous metaplasia and indolent behaviour

**DOI:** 10.1016/j.eucr.2022.102041

**Published:** 2022-02-25

**Authors:** Alessandro Pietro Aldera, Amsha Ramburan, Jeff John

**Affiliations:** a Division of Anatomical Pathology, Faculty of Health Sciences, University of Cape Town, South Africa; b JDW Pathology Inc, Cape Town, South Africa; c National Health Laboratory Service, Groote Schuur Hospital, Cape Town, South Africa; d Division of Urology, Department of Surgery, Frere Hospital and Walter Sisulu University, East London, South Africa

**Keywords:** Renal cell carcinoma, Xp11.2, TFE3, Oncology, Bone

## Abstract

TFE3-rearranged renal cell carcinoma (RCC) is a rare but well characterised histological subtype of RCC with an aggressive clinical course and propensity for late metastases. Osseous metaplasia is an uncommon but well documented finding in clear cell, papillary and chromophobe RCC. We present the first case of a *TFE3*-rearranged RCC to be found harbouring metaplastic bone in a 47-year-old woman who presented with a slowly enlarging left flank mass over a 10 year period. This case report adds to the clinicopathological description of *TFE3*-rearranged RCC and suggests that larger studies are required to fully elucidate the prognosis of these tumours.

## 1 Introduction

Renal cell carcinoma (RCC) is one of the leading causes of cancer worldwide, accounting for approximately 4% of new cancer diagnoses in 2021. *TFE3*-rearranged RCC belongs to the MiT family translocation RCC group and is uncommon, representing 1.6–4% of all cases of RCC in adults.^1^ The overall survival of patients with *TFE3*-rearanged RCC is poor, similar to those with clear cell RCC (CCRCC), and significantly worse than those with papillary RCC (PRCC). *TFE3*-rearranged RCC may metastasise late, up to 20–30 years after diagnosis, necessitating long term clinical follow up. Osseous metaplasia in renal tumours is an uncommon but well described phenomenon with several reports occurring in CCRCC, PRCC and chromophobe RCC. Two cases of *TFE3*-rearanged RCC with osseous metaplasia are described in the literature, one of which was confirmed with RT-PCR.^2^^,^^3^

We present the first case of TFE3-rearranged RCC harbouring osseous metaplasia to be confirmed with fluorescent in situ hybridisation (FISH), with an indolent clinical course and long interval period between presentation and definitive surgery.

## 2 Case presentation

**Fig. 1:**
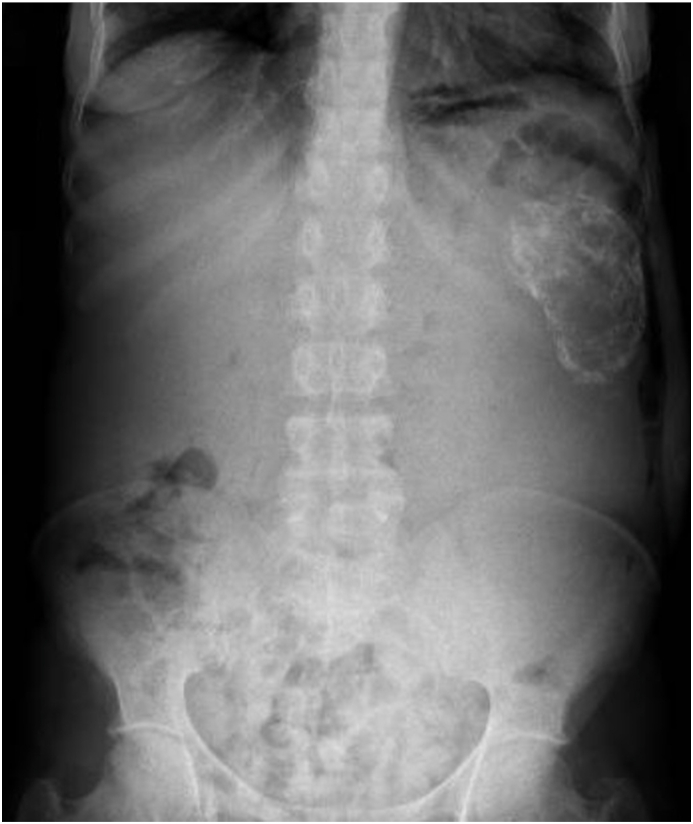
Abdominal X-ray showing a calcified lesion in the left upper quadrant.

**Fig. 2:**
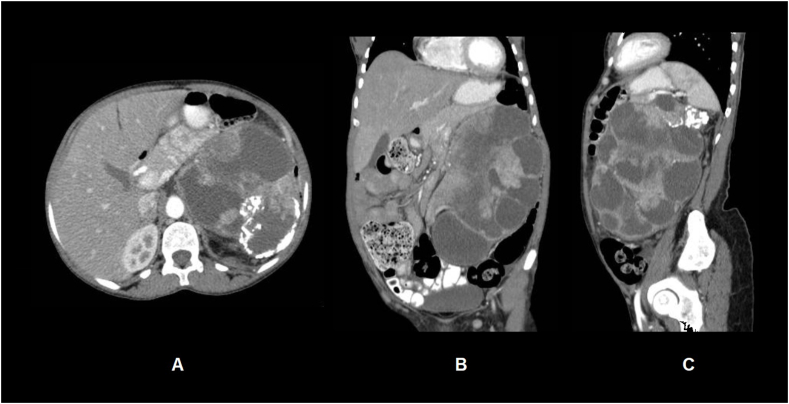
Axial (A), coronal (B) and sagittal (C) views of the computed tomography of the abdomen show a large heterogenous mass of the left kidney with solid, cystic, and calcific components.

A 47-year-old female, known HIV-positive (CD4 312 cells/uL), was referred to our unit with worsening abdominal discomfort and a progressively enlarging left flank mass. Further history revealed that the patient was diagnosed with "kidney cancer" at another hospital ten years ago. She had declined surgery then and had returned now due to worsening symptoms. On examination, vital signs were normal, and no haematuria was noted on urinalysis. The abdominal examination noted a large, firm, smooth, but mobile left flank mass that appeared to extend medially across the midline and caudally into the pelvis. Full blood count and renal function tests were within normal range. An abdominal x-ray revealed the presence of a calcified lesion in the left upper quadrant (Fig. 1). Computed tomography (CT) confirmed the presence of a sizeable heterogeneous left renal mass (22.1 x 14.4 x 13.7 cm) with solid, cystic, and calcific components (Fig. 2). The mass appeared to cross the midline, with displacement of the aorta. No involvement of the renal vein and inferior vena cava was noted. No locoregional extension of the mass into adjacent structures or metastatic lesions were identified. A tentative diagnosis of RCC was made, and the patient underwent a left radical nephrectomy via an anterior subcostal approach. No apparent lymph nodes were identified intraoperatively.

**Fig. 3:**
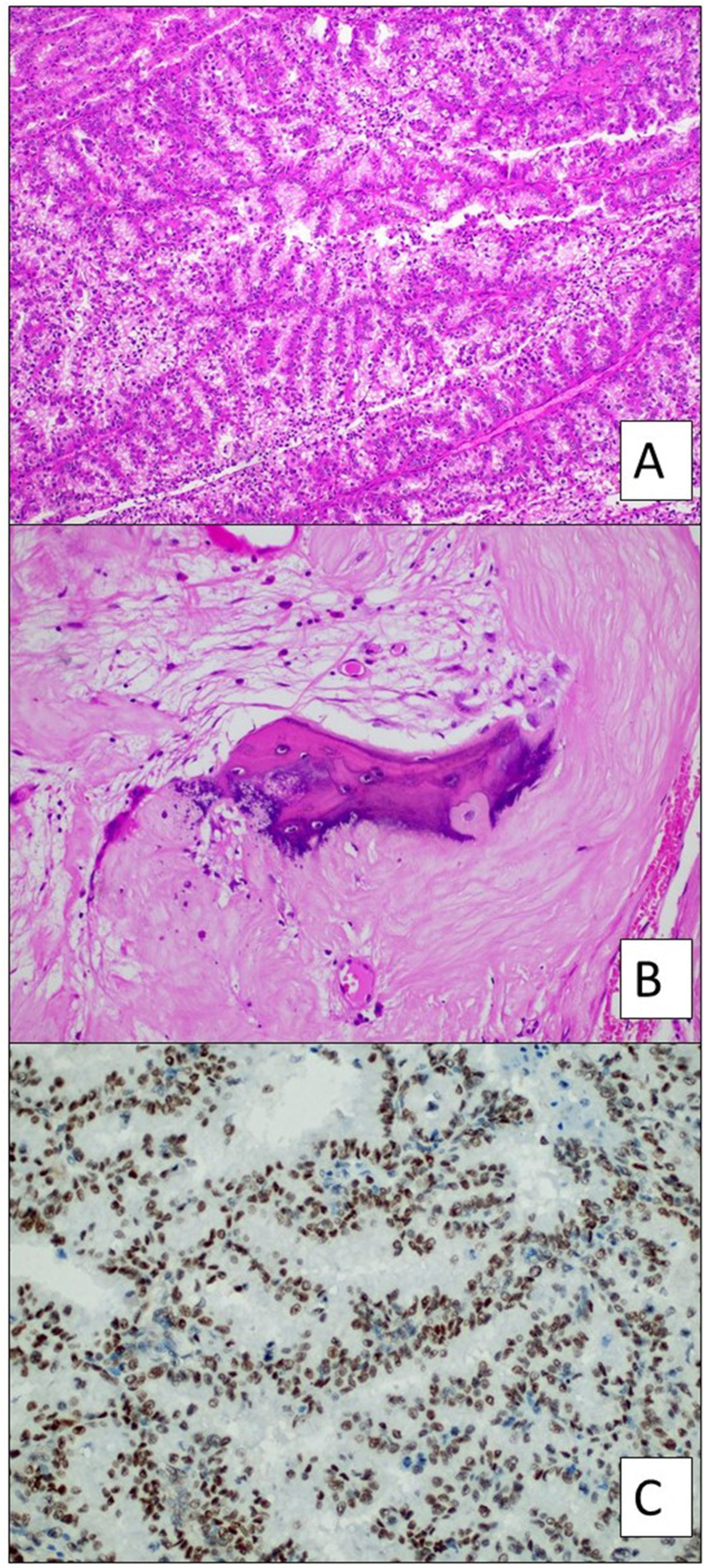
(A) Papillary architecture and prominent apical cytoplasmic clearing are features at low power magnification (Haematoxylin and Eosin (H&E), 100x magnification). (B) Trabeculae of mature bone are seen within the fibrous tumour capsule (H&E, 200x magnification). (C) TFE3 immunohistochemistry shows strong and diffuse nuclear labelling (200x magnification).

Gross examination revealed a large multinodular bosselated tumour which had engulphed most of the kidney. Upon sectioning, a think fibrous and calcified capsule was noted. The tumour comprised multilocular cysts, some of which contained papillary structures, as well as solid areas, with extensive necrosis. The tumour involved the renal sinus (pT3a). Histological examination demonstrated RCC with papillary, pseudopapillary, solid and cystic architecture. The tumoural cells contained moderate eosinophilic cytoplasm in areas, but other areas showed apical cytoplasmic clearing (Fig. 3a). Nucleoli were inconspicuous at 100x magnification (WHO/ISUP grade 2). The fibrous capsule contained extensive dystrophic calcification and areas of ossification (Fig. 3b). Immunohistochemistry showed strong and diffuse positive staining with PAX8, Carbonic anhydrase 9, racemase and TFE3 (Fig. 3c). CK7 was negative. FISH using a *TFE3* dual colour break apart probe confirmed rearrangement in the *TFE3* gene.

Our patient's postoperative course proved uneventful. She was discharged on the fifth day and remained asymptomatic and tumour-free at the last follow-up six months later.

## 3 Discussion

*TFE3*-rearranged RCC is a relatively recently described histological subtype of RCC which is known to have a poor prognosis, similar to that of CCRCC.^1^ This tumour represents a significant proportion of paediatric RCCs (40%) but is relatively uncommon in adults (1.6–4%). The 10 year interval from initial presentation to definitive surgical intervention in our genetically confirmed case is rather unusual and, to our knowledge, not described in the literature. Long term follow-up data on *TFE3*-rearranged RCC is limited in the adult population, with only a few small case series, and perhaps not entirely representative of the clinical course.

The diagnosis of *TFE3*-rearranged RCC should be suspected histologically in the presence of typical morphology (papillary architecture, polygonal cells with pale to clear cytoplasm and psammoma bodies) and strong diffuse nuclear TFE3 immunoreactivity. The papillary architecture and prominent cytoplasmic clearing may prompt the unwary pathologist to consider PRCC and CCRCC respectively. It is important to distinguish *TFE3*-rearranged RCC from its histological mimics as it has a poor prognosis. This can be achieved with TFE3 FISH or immunohistochemistry. The sensitivity and specificity of TFE3 immunohistochemistry is good, but due to difficulties in optimising the antibody, FISH is generally more reliable on formalin-fixed paraffin-embedded tissue samples.^4^

Osseous metaplasia is an uncommonly reported finding in RCC, but has been documented in several cases of CCRCC, PRCC and chromophobe RCC. There is controversy about whether the presence of osseous metaplasia has any clinical significance or holds any prognostic value. Some authors suggest a favourable prognosis, while others suggest that this histological finding is associated with more high grade tumours and resemble a poor prognosis.^5^ It is also imperative that a clear distinction is made between osseous metaplasia and sarcomatoid carcinoma with bone formation.^5^ The latter is associated with a much poorer prognosis. Two cases of *TFE3*-rearranged RCC with osseous metaplasia have been reported in the literature, one in an elderly woman and one in a teenager.^2^^,^^3^ The only other genetically confirmed case (RT-PCR) was reported by Kuroda et al., occurring in a 73-year-old woman.

## 4 Conclusion

*TFE3*-rearranged RCC is an uncommon histological subtype of RCC in adults, with an unfavourable prognosis, and requires a high index of suspicion on the part of the pathologist to correctly identify. TFE3 immunohistochemistry is difficult to optimise, and equivocal or patchy positive staining should prompt genetic confirmation of the translocation with FISH. Osseous metaplasia is uncommon in RCC, and this is the second description of this phenomenon in a genetically confirmed *TFE3*-rearranged RCC. We present a case of *TFE3*-rearranged RCC with indolent behaviour and an uneventful 10 year pre-operative course, suggesting that larger studies focusing on follow-up and outcome data are required to establish a clearer picture of the prognosis of these uncommon tumours.

## Funding

None.

## Declaration of competing interest

The authors declare no conflicts of interest.

## References

[1] Sukov W.R., Hodge J.C., Lohse C.M. (2012). TFE3 rearrangements in adult renal cell carcinoma: clinical and pathologic features with outcome in a large series of consecutively treated patients. Am J Surg Pathol.

[2] Zondo M., Mukendi A.M., Dhoodhat F., Mtshali N., Mosiane P. (2021). Xp11. 2 translocation renal cell carcinoma with osseous metaplasia. J Clin Urol.

[3] Kuroda N., Katto K., Tanaka Y. (2010). Diagnostic pitfall on the histological spectrum of adult-onset renal carcinoma associated with Xp11. 2 translocations/TFE3 gene fusions. Med Mol Morphol.

[4] Green W.M., Yonescu R., Morsberger L. (2013). Utilization of a TFE3 break-apart FISH assay in a renal tumor consultation service. Am J Surg Pathol.

[5] Maioli H., Sharma M., Crane G.M., Wu G., Miyamoto H. (2017). Renal cell carcinoma with osseous metaplasia: a case report and literature review. Integr Cancer Ther.

